# Metacognitive therapy for insomnia: An open cohort study on clinical outcomes and the role of cognitive-attentional syndrome factors in insomnia

**DOI:** 10.1016/j.sleepx.2025.100153

**Published:** 2025-09-25

**Authors:** Osame Salim, Serena Bauducco, Annika Norell, Pia Callesen

**Affiliations:** a School of Behavioural, Social and Legal Sciences, Örebro University, Örebro, Sweden; b Cektos - Center for Metakognitiv Terapi, Copenhagen, Denmark

**Keywords:** Insomnia, Metacognitive therapy, Metacognitive, Open trial

## Abstract

**Background:**

Insomnia is a common and distressing disorder. Evidence suggests that metacognitive beliefs and maladaptive strategies, such as worry and rumination, may maintain insomnia.

**Objectives:**

This study is the first trial of metacognitive therapy (MCT) for insomnia. It aimed to evaluate the effects of MCT on insomnia severity and examine how cognitive-attentional syndrome (CAS) components predict these changes.

**Methods:**

An uncontrolled open cohort trial was conducted with 31 participants in a clinic setting. Participants received up to ten MCT sessions and completed weekly measures of insomnia severity, anxiety, depressive symptoms, and metacognitive beliefs, including maladaptive strategies. The primary outcome was remission status from pre-to post-treatment. Associations between components of the cognitive-attentional syndrome (CAS) and insomnia severity were examined using a mixed model. Follow-up data were collected at 3, 6, and 12 months.

**Results:**

At post-treatment, 57 % of participants met remission criteria and 67 % scored below the clinical cut-off. Within-group effect sizes were large for the Insomnia Severity Index (Hedges’ g = 1.64) and moderate for anxiety (HADS-A, g = 0.67) and depression (HADS-D, g = 0.72). Among those providing follow-up data, 87.5 % remained at or further improved their insomnia symptoms by their last assessment. Moreover, both between-person averages and within-person changes in maladaptive CAS strategies significantly predicted ISI scores, whereas only within-person changes in CAS-negative metacognitions reached significance.

**Conclusions:**

These preliminary findings indicate that metacognitive therapy (MCT) shows promise as an intervention for insomnia. However, large-scale, controlled trials are necessary to further evaluate its clinical utility for this condition.

## Highlights

• This is the first treatment trial of metacognitive therapy (MCT) for insomnia.
• Large effects were found on insomnia severity; moderate for anxiety and depression.
• A majority of participants reached remission at post-treatment.
• Changes in CAS strategies and negative metacognitions predicted insomnia change.

## 1 Introduction

Insomnia symptoms are often highly distressing to the individuals’ experiencing them. For most, this problem is temporary but when it persists it can be debilitating and require treatment, current estimates show that insomnia affects approximately 10 % of the global population [1]. A diagnosis of chronic insomnia requires reported difficulties falling asleep, trouble maintaining sleep, or early morning awakenings which occur at least three days per week for three months. In addition, daytime symptoms—such as excessive tiredness or difficulty functioning—must be present. If left untreated, insomnia tends to have a chronic trajectory in about 40 % of patients [1].Figure 2

Cognitive behavioral therapy for insomnia (CBT-I) is the gold-standard treatment recommended by most international guidelines [2]. However, many individuals do not respond to therapy: currently, about 40–60 % improve, depending on criteria used [3,4], whereas around 7 % drop out early and between 14 % and 40 % fail to complete the entire treatment [5]. It is not fully known what distinguishes those that either drop out or do not improve following CBT-I treatment but some research points to high comorbid depressive symptoms as a predictor of treatment dropout [5,6].

Given that a proportion of patients do not improve following CBT-I, researchers have called for efforts to enhance treatment efficacy for insomnia [7]. One promising avenue is to develop treatments that address the significant comorbidity often present in insomnia. Approximately 50 % of those diagnosed with insomnia have some comorbidity with depressive or anxiety disorders [8]. Transdiagnostic treatments may therefore be especially relevant, as a single intervention can target multiple core complaints, potentially reducing dropout rates and improving overall recovery outcomes.

One such transdiagnostic treatment is Metacognitive Therapy (MCT) which is based on the Metacognitive Model of mental disorders [9]. The main principles of MCT are that mental disorders are maintained by excessive and repetitive engaging in rumination, worry, threat monitoring and other maladaptive coping strategies. These activities are collectively known as Cognitive Attentional Syndrome (CAS) and are in turn upheld by metacognitive beliefs which direct attention to internal processing and continued CAS activity. The most common metacognitive beliefs indicated in mental disorders are negative beliefs about uncontrollability and harmfulness of worry and rumination (e.g “I am unable to control my worry” or “Worry is harmful to my brain”). Such maladaptive metacognitions are believed to be shared by most, if not all, disorders, with only slight variations proposed for the specific metacognitions in each diagnostic category [10].

MCT has shown promising results in treating depressive and anxiety disorders. A recent review concluded that MCT was significantly more efficacious than various forms of Cognitive Behavioural Therapy at post-treatment and follow-up [11]. These results were particularly pronounced for anxiety disorders while less strong for major depressive disorders, although still significant. Given that MCT specifically targets worry and rumination [9], key processes implicated in the development and maintenance of insomnia disorder [12,13], its demonstrated efficacy across disorders marked by these features suggests that it may also hold promise for effectively treating insomnia. While Cognitive Behavioral Therapy for Insomnia (CBT-I) and cognitive models of insomnia have long emphasized cognition, arousal, and safety behaviours; the potential contribution of MCT is to focus specifically on metacognitive processes (beliefs about thinking and patterns of attentional engagement) and to reduce CAS activity without prescribing sleep behavior changes.

While no direct treatment trial of MCT for insomnia exists to date, there have been studies which have examined parts of the metacognitive model in relation to insomnia in different ways. For instance, metacognitive beliefs have been shown to relate specifically to sleep quality for patients with insomnia with higher levels of maladaptive metacognition being cross-sectionally linked to more insomnia symptoms [14], suggesting that dysfunctional metacognitive processes may play a role in the maintenance of sleep difficulties. Additionally, patients undergoing CBT-I for their insomnia symptoms showed a reduction in their maladaptive metacognitions following treatment, although a significant proportion of patients continued to exhibit clinically elevated metacognitive beliefs post-treatment [15]. This finding implies that standard CBT-I may not fully address the underlying metacognitive processes that contribute to insomnia. Other studies on concepts similar to metacognition, such as dysfunctional beliefs in insomnia and unhelpful cognitive strategies have demonstrated that these function both as predictors for incident and persistent insomnia as well as mediators of CBT-I [16,17]. These results taken together point to the potential relevance of metacognition in insomnia as well as the interest in examining the effects of MCT in a treatment trial.

This open label cohort trial study therefore aims to evaluate the effects of individual MCT on adult patients with insomnia on their self-reported sleep difficulties in a private clinic setting. The secondary aims are to investigate the effects of MCT on co-occurring symptoms of anxiety and depression as well as examine whether changes in metacognitive beliefs and maladaptive strategies predict symptom improvement.

## 2 Methods

### 2.1 Design

This study is an open label cohort trial study investigating the treatment effects of MCT for insomnia. The trial period lasted from early 2020 to autumn 2023 during which the final follow-up measures were collected. The study was initially designed to be a randomized controlled pilot trial but following issues with the covid-19 pandemic and difficulties retaining participants in the waitlist condition the trial was redesigned into a one arm cohort study with participants being treated as enrolled. Weekly measures of primary, secondary and process outcomes were administered under the treatment period and at 3, 6 and 12 months following post-treatment. This trial was preregistered at ISRCTN registry (ISRCTN85892563). Reporting followed STROBE checklist guidelines [18], the full checklist is available via Supplemental Material 1.

The setting was a private therapy clinic in Copenhagen, Denmark which offers metacognitive therapy to individuals at self-cost, the study participants were offered the treatment free of charge. Following completion of the data collection a collaboration was established with Örebro university in 2024 to analyze and report the findings.

### 2.2 Participants

Recruitment was made through advertisements on the clinic website. Interested individuals were screened for eligibility after application and given information regarding the trial both verbally and in writing. Criteria for inclusion were being aged between 18 and 70, having a score of at least 11 on the Insomnia Severity Index at the time of intake, as well as receiving a clinical diagnosis of insomnia in our screening assessment. The criteria for exclusion were previous treatment with metacognitive therapy, concurrent psychological treatment, or recent alterations in medicinal treatment for insomnia. Participants were also excluded if they suffered from severe depression with suicidal intent, psychosis, personality disorders, or current substance abuse. Additionally, participants were excluded if they had sleep-disruptive medical illnesses driving their current symptoms (See Table 1).

**Table 1:** Participant characteristics.

Variable		M (SD)	% (n)
Age (years)		47.39 (10.92)	
Gender (female)			80.6 (25)
Civil status	Single		40.7 (11)
	Cohabitating/Married		59.2 (16)
Highest completed education level	High school		3.5 (1)
	University		96.4 (27)
	Missing		9.7 (3)
Occupational status	Employed		64.2 (18)
	Student		10.7 (3)
	Sick leave		7.1 (2)
	Pensioner		14.3 (4)
	Unemployed		3.6 (1)
	Missing		9.7 (3)
Previous psychological treatment	No		21.4 (6)
	Yes		78.6 (22)
	Missing		9.7 (3)
Concurrent medication use	Sleep medication		24.0 (6)
	Other psychiatric medication		12.0 (3)
	Other somatic medication		20.0 (5)
	No medication		44.0 (11)
	Missing		19.3 (6)

A total of 50 individuals self-referred to the trial via application on the clinic website. From these, 33 participants were able to be reached for a screening assessment which used the Structured Clinical Interview for DSM-IV-Axis I Disorders (SCID-I). SCID-I is a semi-structured interview guide for the major DSM-IV Axis I diagnoses. The SCID-I has an inter-rater reliability of between 0.61 and 0.83 with a mean of 0.71 [19]. The SCID-was used solely to determine whether participants met our inclusion/exclusion thresholds. We used SCID-I for pragmatic reasons: at study start, a validated Danish-language SCID-5 instrument was not available in the clinic, clinicians were trained on SCID-I, and our eligibility decisions were anchored in ISI thresholds and the clinical insomnia presentation. We did not code all SCID sections, and so cannot report primary versus secondary insomnia rates, chronicity of symptoms, or full psychiatric comorbidity profiles beyond the study's exclusion criteria.

During the same interview participants were asked (yes/no) about any prior psychotherapy for sleep or other mental health problems and whether they were currently taking any prescription medications for sleep or other conditions. No further details on treatment modality or dosing were recorded. Consistent with our protocol, all participants were included only if their medication regimens were stable before and throughout the 10-week MCT trial.

**Fig. 1:**
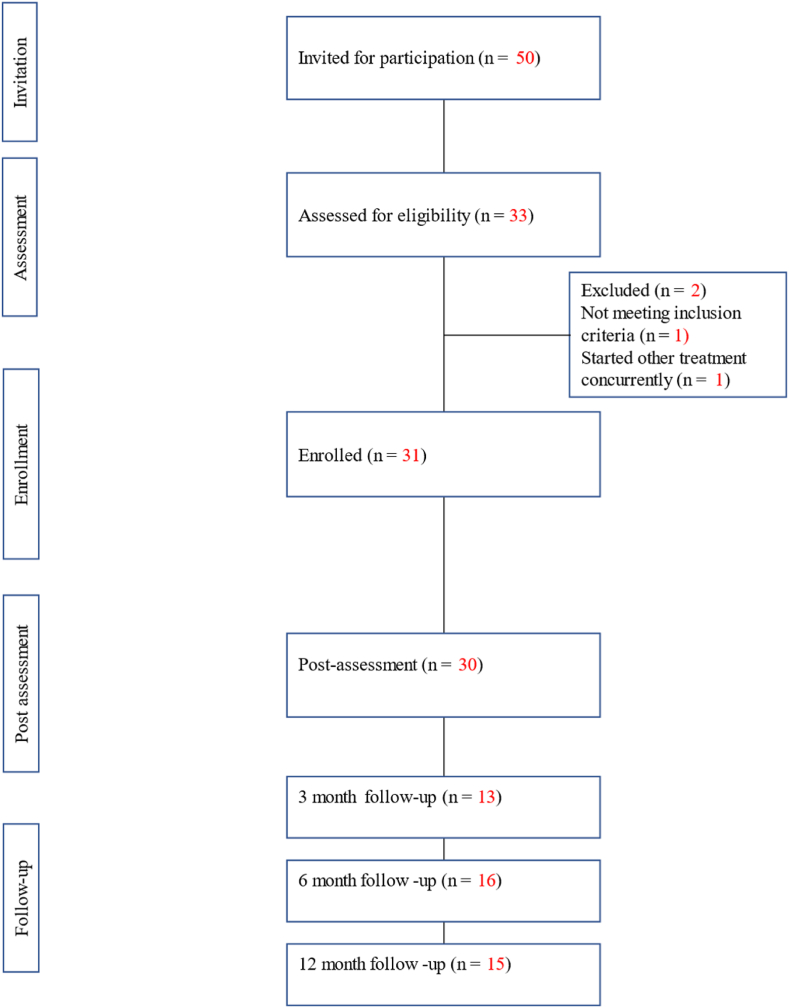
CONSORT flow diagram of participant progress.

Out of the 33 participants screened, one did not meet the inclusion criteria, and one additional participant was excluded due to starting another psychological treatment after the screening. No participant dropped out during treatment but for one participant the post assessment form was not collected due to a handling error (See Fig. 1).

### 2.3 Ethical considerations

The study was registered with the Danish Ethics Committee and DSAM (Sags ID: EMN-2020-35209). Data collection and storage adhered to GDPR regulations. The participants who were patients at the clinic, “Cektos– Center for Metakognitiv Terapi”, were insured by Tryg Insurance as part of Cektos routine treatment coverage. Informed consent was obtained from all participants before enrollment, with both verbal and written information provided at time of screening. Participants were informed that they could withdraw from the study at any time without any consequences. A protocol was established for monitoring and managing both adverse events (AEs) and serious adverse events (SAEs) during the trial. Adverse events were defined as any unintended effects, such as side effects or worsening of symptoms. Serious adverse events included situations such as death, life-threatening conditions, hospitalization, or significant disability. In the event of an SAE, the research team would immediately contact appropriate medical services and notify the ethical committee. All therapists were licensed mental health professionals trained to assess client safety and well-being. No adverse or serious adverse events were reported during the trial period.

### 2.4 Measures

Demographic data was collected from the participants in the study. This included age, gender, civil status, highest level of education completed, employment status, concurrent medication use, and previous psychological treatment (coded as a binary variable).

The Insomnia Severity Index (ISI) was employed to evaluate participants' overall perception of insomnia severity [4,20]. This 7-item questionnaire—featuring items such as “How satisfied are you with your current sleep pattern?”—is rated on a 5-point scale (0–4), yielding a total score ranging from 0 to 28 with higher scores indicating greater insomnia symptoms. The Cronbach's alpha of the ISI was reported as 0.83 in a recent meta-analysis [21]. In the current sample, internal consistency was lower (Cronbach's α = 0.60).

The Hospital Anxiety and Depression Scale (HADS [22]; was used to assess symptoms of anxiety and depression. The HADS consists of 14 items divided into two subscales: anxiety (HADS-A) and depression (HADS-D), each comprising seven items rated on a 4-point Likert scale (0–3). For example, one HADS-A item states, “I feel tense or wound up.” The subscale scores range from 0 to 21, with higher scores indicating greater symptom severity. The HADS has demonstrated good internal consistency, with Cronbach's alpha of 0.83 reported in a literature review [23]. In the present sample, internal consistency was higher (Cronbach's α = .88).

The Cognitive Attentional Syndrome-1 (CAS-1) scale was used to measure changes in metacognitive beliefs and processes. This scale is based on the Metacognitive model and assesses both positive (e.g., “my worry helps me cope”) and negative (e.g., “my worry is uncontrollable”) metacognitive beliefs, along with maladaptive coping strategies such as rumination, avoidance, and threat monitoring [10]. A large-scale psychometric study found the CAS-1 to have a good internal consistency with Cronbach's alpha ranging from 0.77 to 0.89 [24]. In the present sample, internal consistency was somewhat lower (Cronbach's α = .75).

### 2.5 Procedure

All therapy sessions were provided individually, either in physical form or via live video meeting. The covid-19 pandemic meant that some therapies switched between in-person and video meeting during the course of treatment, sometimes with brief notice if either part showed symptoms. We did not record the percentage of sessions delivered in each format. The participants were given up to ten treatment sessions, if deemed necessary. Any decision to finish treatment before ten sessions was based on consensus between the therapist and client that insomnia was no longer an issue for the client, as well as supported by assessed ISI scores. Participants attended between 3 and 10 sessions with a median of 7 sessions, mode of 5 sessions and a mean of 7.03 (SD = 2.20).

#### 2.5.1 Intervention

The MCT treatment for insomnia in this study adheres to the standardized model and interventions as outlined in the treatment manual by Wells [10]. The main goal of MCT is to reduce the Cognitive Attentional Syndrome (CAS). This syndrome involves a rigid focus on internal threatening stimuli, accompanied by excessive worry, rumination, and maladaptive control strategies aimed at problem-solving insomnia itself. Participants undergo up to 10 weekly sessions of individual MCT, which last for 45 min. The initial session involves a collaborative case formulation, which serves as the foundation for subsequent interventions. CAS is framed as the primary maintaining factor of patients' insomnia, and goals of the treatment are established. After case formulation, detached mindfulness (DM) is introduced as a direct counter to the Cognitive Attentional Syndrome (CAS). Subsequently, patients' negative metacognitive beliefs about the uncontrollability of worry and rumination are addressed through verbal reattribution strategies. This is done using questions such as, “Have you had any experiences where you were able to leave triggering thoughts alone?” or “What evidence do you have that you can let thoughts pass while lying in bed?” These beliefs are further targeted through behavioral experiments, such as the association task. In this task, the therapist reads aloud a series of neutral and then triggering words, while the patient practices allowing any mental associations to arise and pass without engagement. Once uncontrollability beliefs have been sufficiently challenged, the therapist proceeds to address metacognitive danger beliefs. These are similarly explored and tested through a combination of verbal reattribution and behavioral experiments which aim to test and disprove beliefs of danger. Lastly, patients' positive metacognitive beliefs such as the helpfulness and usefulness of worry about one's sleep and monitoring your body for sleep-related cues. In the final sessions, relapse prevention work was conducted by helping the patient develop a more adaptive “new plan.” This plan emphasized the use of detached mindfulness in response to triggering thoughts, in contrast to the patient's previous “old plan,” which relied on maladaptive cognitive strategies such as worry, excessive planning, and thought suppression. Finally, participants are informed about the expected times for follow-up at the last session. Importantly, and in line with study aims, the intervention did not include CBT-I components such as sleep diaries, sleep restriction, stimulus control, relaxation training, psychoeducation about sleep, or cognitive restructuring related to sleep.

#### 2.5.2 Therapists and supervision

Five therapists, four female and one male, provided the treatment in this trial. All were clinical psychologists and licensed mental health providers with a minimum of master's degree in psychology. Clinicians had between 0 and 4 years of clinical experience after licensure with a mean of 1.4 (SD = 1.5) years of clinical experience. All therapists had either completed MCT masterclass level 1 from Metacognitive Therapy Institute (MCTI) or were undergoing training during the period of the trial. In addition, all therapists underwent monthly supervision, lasting approximately 90 min. The supervisor was a level 2 MCTI ® registered therapist with experience in providing supervision and training in MCT. The supervision meetings were in group and contained video and oral presentation of cases. A competency and adherence scale developed for MCT, Metacognitive Therapy Competency Scale (MCT-CS), was used to assess intervention fidelity during supervision meetings.

### 2.6 Data analysis

#### 2.6.1 Missing data

The missing data overall in this trial varied across different timepoints between 13 and 24 % for the ISI, 26–47 % for the HADS, and 13–45 % for CAS-1 measures. The reasons for missingness were that the therapists had misinterpreted instructions regarding the administration of measures (at which time points, and which scales) as well as failures of data handling such as scanning the first page of the measures and not the second page of the measures. As such, the missing data is not presumed to be related to their expected true value and can be handled under the Missing at Random (MAR) assumption [25] For three participants with missing baseline ISI, week 1 assessments were used as a proxy to enable pre–post evaluation. As a sensitivity check, we repeated analyses excluding the three participants whose baseline ISI was substituted with week-1 values. Results were unchanged in terms of significance and direction. Of the total sample, 77 % (n = 24) of the participants provided at least one follow-up assessment. Non-response rates varied by time point, ranging from 58 % to 61 % at the 3-month follow-up, 48 %–52 % at 6 months, and 52 %–61 % at 12 months, depending on the measure (see Supplemental Material 2.). In contrast to during treatment the reasons for missingness in the follow-up period are plausibly connected to their true value which means that follow-up data were treated as Missing Not at Random (MNAR) and analyzed descriptively only.

#### 2.6.2 Power calculations

No previous studies on the effects of Metacognitive Therapy (MCT) for insomnia existed at the time of trial. The original power calculation aimed at detecting a large effect of MCT in comparison to a waitlist control condition. Assuming an alpha level of 0.05 and a power of 0.8, a two-tailed *t*-test was conducted with an expected effect size of Cohen's d = 0.85 since most MCT treatments show large effects against waitlist controls (Andersson et al., 2025.) Based on these parameters, it was determined that a sample size of 32 participants was required (calculated using G∗Power 3.1.97).

However, since the study was redesigned as an open trial, a within-group power calculation was necessary to detect at least a medium to large effect size of Cohen's d = 0.5. Using the same alpha level of 0.05 and a power of 0.8, it was estimated that a sample size of 34 participants would be required to achieve adequate statistical power.

#### 2.6.3 Clinically significant reliable change, remission, and improvement

The primary outcome was measured using the clinically significant reliable change (CSRC) criteria set by Jacobson & Truax [26]. The reliability estimates for calculating CSRC was taken from a recent meta-analysis of the ISI [21] Remission status was defined as meeting both CSRC criteria and ending treatment in the non-clinical range (i.e., ISI score reduced from above 15 to 11 or less), a cutoff that has been shown to distinguish clinical from non-clinical populations [4]. In addition to CSRC and remission, we also assessed levels of clinical improvement. Moderate improvement was defined as a reduction of 8.36 points or more on the ISI, and slight improvement as a reduction of 4.65 points or more [4]. These thresholds were used to capture varying degrees of clinically meaningful change across participants.

#### 2.6.4 Follow-up data

For the follow‐up period, only descriptive analyses were conducted due to the presumed missing not at random (MNAR) missingness mechanism. Participants who provided at least one follow-up assessment were included in these analyses. Each participant's post-treatment Insomnia Severity Index (ISI) score was compared with their score at the last available follow-up assessment. Changes in ISI scores were categorized as follows: a reduction in score of 4.65 or greater indicated a slight improvement in symptoms, whereas an increase in score of 4.65 or greater was defined as a slight worsening of symptoms [4]. Descriptive statistics (i.e., percentages, means, and standard deviations) were calculated to summarize the ISI scores at the 3-, 6-, and 12-month follow-up time points.

#### 2.6.5 Linear mixed model and model Selection

A linear mixed model was employed, using maximum likelihood estimation, to examine the effect of cognitive attentional syndrome (CAS) on insomnia severity (ISI) from baseline to post-treatment. The CAS-1 measure, which comprises three subscales—negative metacognitive beliefs, positive metacognitive beliefs, and maladaptive cognitive strategies—was decomposed into between-person (mean) and within-person (time-varying) components to differentiate stable individual differences from changes over time [27]. A random intercept for each participant was included. ;

Because the role of metacognition in insomnia treatment is relatively unexplored, we aimed to capture the distinct contributions of positive beliefs, negative beliefs, and strategies from the CAS-1 measure in an exploratory analysis. Initially, we specified a “maximal” model that included random slopes for all within- and between-person predictors; however, this model was overparameterized and did not converge. We followed the recommendations of Bates et al. [28] and applied a stepwise reduction strategy: variance components were estimated for each predictor while constraining its corresponding covariance parameter to zero. Next, we removed any variance component that was zero or negligible, based on likelihood ratio tests (LRTs) and comparisons of Akaike information criterion (AIC) and Bayesian information criterion (BIC) values. Finally, we retained only the random slope for within-person changes in CAS strategies, as it showed a meaningful variance component. This more parsimonious model demonstrated equivalent fit according to LRTs, AIC, and BIC, while avoiding the convergence issues associated with an overparameterized model. Because predictors were tested within one model and analyses were exploratory, no multiple comparisons correction was applied. We note, however, that given the small sample and number of predictors, there remains a risk of Type I error, and results should therefore be interpreted with caution.

## 3 Results

### 3.1 Changes in insomnia, anxiety and depressive symptoms from pre-to post-intervention

**Fig. 2:**
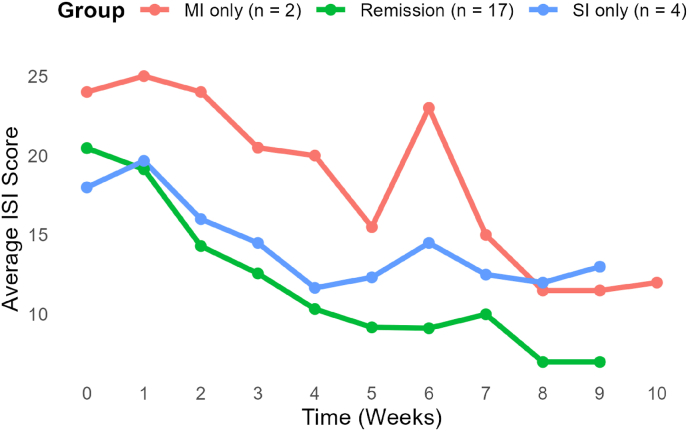
Mean Insomnia Severity Index (ISI) scores from baseline to week 10 across groups of responders. **Note**. N for each curve is the number of participants classified **exclusively** in that group while Table 2 reports the total (overlapping) Ns for each threshold. MI = Moderate Improvement (n = 2); SI = Slight Improvement (n = 4); Remission = remission and CSRC (n = 17). Lower scores reflect fewer insomnia symptoms.

The results showed that 57 % (17 out of 30) of participants achieved clinically significant reliable change (CSRC) and remission status, based on the criteria by Jacobson and Truax (1991) and Morin et al. (2011) for insomnia symptoms measured by the ISI. Additionally, 60 % (18 out of 30) demonstrated moderate improvement, and 77 % (23 out of 30) achieved slight improvement (change of 4.65 or greater) (SeeTable 2; Fig. 2).

**Table 2:** CSRC and outcome scores on the ISI and HADS (N = 30).

Outcome	N (%)	Pre-treatment *M* (SD)	Post-treatment *M* (SD)	Effect Size (*g*)	*p*-value
Remission (CSRC)^a^	17 (57 %)				
Moderate improvement^b^	18 (60 %)				
Slight improvement^b^	23 (77 %)				
ISI total score		19.82 (3.45)	9.80 (4.84)	1.64	<0.001
HADS-Anxiety subscale score		9.59 (4.32)	6.57 (4.13)	0.76	<0.01
HADS-Depression subscale score		5.09 (3.34)	3.14 (3.23)	0.65	<0.01

**Note.** N = 30. ISI = Insomnia Severity Index; HADS = Hospital Anxiety and Depression Scale. Clinically significant reliable change and improvement criteria were based on Jacobson and Truax (1991) and Morin et al. (2011). Effect sizes are reported as Hedges' g. N in table = number of participants who ever met this threshold; groups overlap.

a Defined as meeting criteria for clinically significant reliable change and remission status.

b Defined thresholds for slight (≥4.65-point decrease) and moderate (≥8.36-point decrease) improvement are based on change scores on the ISI.

The mean ISI score decreased from pre-treatment (M = 19.82, SD = 3.45) to post-treatment (M = 9.80, SD = 4.84), which corresponded to a large effect size (Hedges’ *g* = 1.64). Significant improvements were also observed on the Hospital Anxiety and Depression Scale (HADS). Anxiety scores decreased from M = 9.59 (SD = 4.32) to M = 6.57 (SD = 4.13), *g* = 0.76, *p* < .01; and depression scores decreased from M = 5.09 (SD = 3.34) to M = 3.14 (SD = 3.23), *g* = 0.65, *p* < .01. At post-treatment 67 % (20 out of 30) scored below the clinical cut-off (ISI ≥11) [4].

### 3.2 Changes in insomnia symptoms follow-up

Out of the 31 participants, 24 responded to at least one follow-up assessment. Improvement or worsening was defined using the slight improvement criterion of 4.65 points or greater change from post-treatment ISI scores to the last available follow-up assessment. Of the 24 participants, 4 (16.7 %) showed further improvement at follow-up compared to post-treatment scores (M = −6.75, SD = 3.50). 17 participants (70.8 %) showed no significant change, indicating either stability or maintenance of treatment gains (M = −0.94, SD = 2.07). 3 participants (12.5 %) exhibited slight worsening in their ISI scores (M = 7.00, SD = 2.64).

Mean ISI scores at each follow-up point were as follows: 3-month (M = 7.32, SD = 5.40), 6-month (M = 9.62, SD = 7.30), and 12-month (M = 9.08, SD = 5.73). At last available follow-up 54 % (13 out of 24) scored below the clinical cut-off (ISI ≥11) [4].

### 3.3 Cognitive-attentional syndrome components as predictors of insomnia severity

Model fit showed a satisfactory fit with a deviance from an optimal model of 850.5, with AIC = 874.5 and BIC = 911.7. Examination of residuals suggested no major violations of distributional assumptions. The random effects estimates showed notable variability across participants, particularly for the random slope of CAS Strategies (variance = 1.18, SD = 1.09). For the fixed effects, CAS strategies were positively associated with ISI for both between-person mean (Estimate = 2.37, t = 4.32) and within-person change (Estimate = 1.51, t = 4.38) parameters, suggesting that higher average use of CAS strategies—and changes in their use from one's own average—predicted changes in insomnia severity over time. In contrast, neither between-person nor within-person CAS Positive Metacognitions significantly predicted ISI changes, while CAS Negative Metacognitions showed a significant effect for the within-person component (Estimate = 0.08, t = 3.56) but not between-person component (Estimate = −0.04, t = −0.94). Time itself had a negative significant coefficient (Estimate = −0.20, t = −1.58), indicating a decrease in ISI across the study period.

Overall, these findings suggest that within person CAS Negative Metacognitions as well as both stable (between-person) and fluctuating (within-person) levels of CAS strategies are important factors in predicting insomnia severity in this sample.

## 4 Discussion

The primary research question in this open-cohort trial was to examine the effects of Metacognitive Therapy (MCT) for insomnia disorder. The results suggested that 57 % of the treatment sample achieved CSRC and remission in their primary insomnia diagnosis with significant decreases in anxiety and depressive symptoms observed on a group level. Among those providing follow-up data, 87.5 % maintained gains or improved further in insomnia symptoms at their last assessment point. A secondary research question examined the role of cognitive attentional syndrome as a predictor of insomnia improvement. Linear mixed model analysis showed that reductions in negative metacognitions and especially maladaptive strategies, but not positive metacognitions, was associated with improvements in insomnia severity. Indeed, changes in maladaptive strategies predicted insomnia severity after accounting for both between-person (stable individual differences) and within-person (temporal fluctuations) effects. Notably, the significant within-person effects indicate that reductions in maladaptive control and coping strategies over the course of treatment are linked to improvements in insomnia symptoms among participants undergoing MCT for insomnia thus highlighting these as potential mechanisms of change.

### 4.1 Interpretation of results

This study suggests that Metacognitive Therapy (MCT) might be a promising treatment for insomnia. However, randomized controlled trials are necessary to establish its efficacy. Ultimately, direct comparisons between MCT and CBT-I are required to determine relative efficacy. Alternatively, future research should explore whether MCT and CBT-I demonstrate differential efficacy for specific subsets of patients, potentially identifying profiles of individuals who respond better to one treatment over the other. While CBT-I has a robust evidence base targeting behaviors and cognitions that maintain insomnia, MCT differs by focusing on metacognitive processes and CAS reduction without prescribing sleep behavior changes. Future research should investigate whether these approaches are complementary for patients or whether combining them may, in some cases, attenuate treatment effects.

One plausible mechanism of improvement is that MCT reduces perseverative thinking (worry/rumination) and attenuates threat-focused attention and maladaptive monitoring (e.g., scanning for sleep-related cues), thereby lowering cognitive-emotional arousal incompatible with sleep initiation/maintenance [12]. Modifying negative metacognitive beliefs (uncontrollability/danger) may decrease urgency to control thoughts and sleep, reducing counterproductive control strategies (e.g., thought suppression, rigid rituals), which aligns with our findings showing that within person changes in their use of maladaptive strategies are linked to changes in insomnia symptoms.

The sample in this open trial was comprised primarily of female participants who had completed some form of higher education and were predominantly employed. Consequently, these findings may not generalize to populations with fewer socioeconomic resources. Future research should examine the effects of MCT across diverse demographic groups, including traditionally underserved populations such as ethnic minorities and socioeconomically disadvantaged individuals.

These preliminary findings suggest that significant improvements in insomnia severity may be achieved even without directly addressing behavioral sleep patterns through interventions such as sleep education, stimulus control, or sleep restriction. Notably, the MCT protocol does not require patients to modify their sleep behaviors, apart from advising them to stop using maladaptive control strategies, such as rigid rituals like drinking warm milk or excessively focusing on winding down before bed. This contrasts with conventional CBT-I, which typically includes sleep education and behavioral instructions on practices to adopt or avoid to promote better sleep. From the MCT perspective, such instructions may unintentionally maintain an excessive cognitive focus and ruminative efforts toward achieving sleep, thereby exacerbating the problem.

### 4.2 Strengths and limitations

This study has several strengths. Weekly assessments of both outcome and process measures allowed for the investigation of time-dependent relationships and preliminarily examining potential mechanisms of change. The extended 12-month follow-up period provided possible insights into the durability of MCT effects on this population. Additionally, all therapists delivering the intervention were either registered MCT therapists or under training to become registered MCT therapists. The treatment was provided under supervision of a Level 2 MCT therapist with extensive experience in training and supervision which provided good conditions for treatment fidelity and purity. Additionally, we evaluated the clinical significance on an individual level (response and remission) using well-established cutoffs for the primary outcome measure of ISI.

However, this study is not without limitations. The absence of a control group prevents full control for potential maturation and history effects, although previous research has noted that insomnia tends to become chronic without intervention for circa 40 % of individuals (Morrin & Jarrin, 2022). Future research should build on these preliminary findings by conducting randomized controlled trials with active and passive control conditions.

Additionally, in the current study the potential for bias due to missing data warrants consideration. In our primary analysis of CSRC and remission, missing data was limited to 1 of 31 cases in the pre-to post-assessments, indicating a relatively low impact on the main findings. As a secondary analysis, a linear mixed model (LMM) with full information maximum likelihood estimation was employed to account for the missing data. Additionally, the LMM was based on a small sample and included several correlated predictors, raising some risk of Type I error emphasizing the need for replication in adequately powered, controlled trials.

The follow-up data were not included in any formal statistical analysis, as they are presumed to follow a MNAR mechanism, where the likelihood of missingness may be related to participants’ underlying values. As such, although the direction of potential bias is uncertain, all results from follow-up analyses should be interpreted descriptively.

Finally, reliance on only self-report without objective sleep measures limits interpretation of changes in actual sleep performance. Future potential trials of MCT for insomnia should consider incorporating objective measures —such as actigraphy, polysomnography—to enhance the study's rigor. However, given that data collection for this study occurred in a private clinical setting with limited resources for additional equipment, such measures were not feasible at the time.

### 4.3 Future directions

The finding that negative metacognitions and maladaptive strategies predict subsequent improvements in symptom severity aligns with the metacognitive theoretical model as well as the broader research findings on the effects of worry and rumination on sleep [12]. However, this study employed a generic MCT treatment model with only slight adjustments made to the insomnia presentation. Future research should investigate which specific metacognitions are most central to insomnia. The development of an insomnia-specific MCT model could help refine treatment protocols and hopefully boost treatment effects. Further studies should also aim to specify the causal relationship between negative metacognitions, positive metacognitions, and maladaptive strategies through experimental work or carefully designed observational studies aimed to support causal interpretations [29].

We used remission and CSRC as our primary analysis of outcome, which allows for an assessment of individual-level treatment response. However, the percentage of participants achieving clinically significant change is sensitive to sample size, and larger trials will likely provide more accurate population estimates. Additionally, remission in this study was based on two criteria [1]: meeting the cut-off defined by Morin et al. (2011) for the clinical population, and [2] fulfilling CSRC status. Other studies have used different, sometimes more lenient, definitions of response and remission [3], limiting comparability across trials. Future research should establish standardized guidelines for defining remission status to improve consistency across studies and facilitate comparisons of treatment effects.

### 4.4 Conclusion

In conclusion, this study represents the first investigation of metacognitive therapy (MCT) for insomnia, with findings suggesting promising treatment effects. The regression model provided support for the cognitive attentional syndrome framework in predicting insomnia symptom levels and treatment-related changes. Although encouraging, these results should be interpreted with caution given the study's lack of control group. Future research with controlled designs is warranted to further evaluate the clinical utility of MCT as an alternative intervention for insomnia and to potentially enhance access to effective treatment for individuals suffering from this condition.

## CRediT authorship contribution statement

**Osame Salim:** Writing – review & editing, Writing – original draft, Visualization, Validation, Software, Resources, Formal analysis. **Serena Bauducco:** Writing – review & editing, Supervision, Methodology. **Annika Norell:** Writing – review & editing, Supervision, Project administration. **Pia Callesen:** Writing – review & editing, Resources, Project administration, Methodology, Investigation, Data curation, Conceptualization.

## Declaration of generative AI and AI-assisted technologies in the writing process

During the preparation of this work the authors used GPT 4o and o3-mini in order to improve language and readability. After using this tool/service, the authors reviewed and edited the content as needed and takes full responsibility for the content of the publication.

## Funding

The author OS received a short-term mobility grant for PhD students from Erasmus to support travel and housing during data collection. No additional funding sources are declared.

## Declaration of competing interest

The authors declare the following financial interests/personal relationships which may be considered as potential competing interests:The author Pia Callesen owns and operates a private clinic that provides treatment and training in Metacognitive Therapy and has authored books on Metacognitive Therapy. No other conflicts of interest are declared.

## References

[1] Morin C.M., Jarrin D.C. (2022 Jun 1). Epidemiology of insomnia: prevalence, course, risk factors, and public health burden. Sleep Med Clin.

[2] Riemann D., Baglioni C., Bassetti C., Bjorvatn B., Dolenc Groselj L., Ellis J.G. (2017). European guideline for the diagnosis and treatment of insomnia. J Sleep Res.

[3] Davidson J.R., Dawson S., Krsmanovic A. (2019 Mar 4). Effectiveness of group cognitive behavioral therapy for insomnia (CBT-I) in a primary care setting. Behav Sleep Med.

[4] Morin C.M., Belleville G., Bélanger L., Ivers H. (2011 May 1). The insomnia severity index: psychometric indicators to detect insomnia cases and evaluate treatment response. Sleep.

[5] Ong J.C., Kuo T.F., Manber R. (2008 Apr). Who is at risk for dropout from group cognitive-behavior therapy for insomnia?. J Psychosom Res.

[6] Yeung W.F., Chung K.F., Ho F.Y.Y., Ho L.M. (2015 Oct). Predictors of dropout from internet-based self-help cognitive behavioral therapy for insomnia. Behav Res Ther.

[7] Manber R., Simpson N., Gumport N.B. (2023 Oct 31). Perspectives on increasing the impact and reach of CBT-I. SLEEP.

[8] Morin C.M., Bertisch S.M., Pelayo R., Watson N.F., Winkelman J.W., Zee P.C. (2023 Mar 2). What should be the focus of treatment when insomnia disorder is comorbid with depression or anxiety disorder?. J Clin Med.

[9] Wells A. (2019 Dec 12). Breaking the cybernetic code: understanding and treating the human metacognitive control system to enhance mental health. Front Psychol [Internet].

[10] Wells A. (2009).

[11] Andersson E., Aspvall K., Schettini G., Kraepelien M., Särnholm J., Wergeland G.J. (2025 Mar 4). Efficacy of metacognitive interventions for psychiatric disorders: a systematic review and meta-analysis. Cogn Behav Ther.

[12] Clancy F., Prestwich A., Caperon L., Tsipa A., O'Connor D.B. (2020 Oct). The association between worry and rumination with sleep in non-clinical populations: a systematic review and meta-analysis. Health Psychol Rev.

[13] Fröjd L.A., Papageorgiou C., Munkhaugen J., Moum T., Sverre E., Nordhus I.H. (2022). Worry and rumination predict insomnia in patients with coronary heart disease: a cross-sectional study with long-term follow-up. J Clin Sleep Med.

[14] Palagini L., Piarulli A., Menicucci D., Cheli E., Lai E., Bergamasco M. (2014 Aug). Metacognitive beliefs relate specifically to sleep quality in primary insomnia: a pilot study. Sleep Med.

[15] Galbiati A., Sforza M., Scarpellino A., Salibba A., Leitner C., D'Este G. (2021 Sep 9). “Thinking About Thinking” in insomnia disorder: the effect of cognitive-behavioral therapy for insomnia on sleep-related metacognition. Front Psychol.

[16] Parsons C.E., Zachariae R., Landberger C., Young K.S. (2021 Jun 1). How does cognitive behavioural therapy for insomnia work? A systematic review and meta-analysis of mediators of change. Clin Psychol Rev.

[17] Norell-Clarke A., Hagström M., Jansson-Fröjmark M. (2021 Jun 21). Sleep-related cognitive processes and the incidence of insomnia over time: does anxiety and depression impact the relationship?. Front Psychol [Internet].

[18] von Elm E., Altman D.G., Egger M., Pocock S.J., Gøtzsche P.C., Vandenbroucke J.P. (2008 Apr). The strengthening the reporting of observational studies in epidemiology (STROBE) statement: guidelines for reporting observational studies. J Clin Epidemiol.

[19] Lobbestael J., Leurgans M., Arntz A. (2011). Inter-rater reliability of the structured clinical interview for DSM-IV axis I disorders (SCID I) and axis II disorders (SCID II). Clin Psychol Psychother.

[20] Bastien C.H., Vallières A., Morin C.M. (2001 Jul 1). Validation of the insomnia severity index as an outcome measure for insomnia research. Sleep Med.

[21] Cerri L.Q., Justo M.C., Clemente V., Gomes A.A., Pereira A.S., Marques D.R. (2023 Aug). Insomnia severity index: a reliability generalisation meta-analysis. J Sleep Res.

[22] Zigmond A.S., Snaith R.P. (1983). The hospital anxiety and depression scale. Acta Psychiatr Scand.

[23] Bjelland I., Dahl A.A., Haug T.T., Neckelmann D. (2002 Feb). The validity of the hospital anxiety and depression scale. An updated literature review. J Psychosom Res.

[24] Nordahl H., Wells A. (2019 Dec). Measuring the cognitive attentional syndrome associated with emotional distress: psychometric properties of the CAS-1. Int J Cognit Ther.

[25] Enders C.K. (2022).

[26] Jacobson N.S., Truax P. (1991). Clinical significance: a statistical approach to defining meaningful change in psychotherapy research. J Consult Clin Psychol.

[27] Wang L., Peggy, Maxwell S.E. (2015). On disaggregating between-person and within-person effects with longitudinal data using multilevel models. Psychol Methods.

[28] Bates D., Kliegl R., Vasishth S., Baayen H. (2018). Parsimonious mixed models [internet]. arXiv.

[29] Rohrer J.M. (2018 Mar). Thinking clearly about correlations and causation: graphical causal models for observational data. Adv Methods Pract Psychol Sci.

